# Psychotherapy Style Scale: Development and Validation

**DOI:** 10.3389/fpsyg.2021.543989

**Published:** 2021-05-24

**Authors:** Shegang Zhou, Yanfei Hou, Ding Liu, Duo Xu, Xiaoyuan Zhang

**Affiliations:** ^1^Department of Psychology, Institute of Education, Henan Normal University, Xinxiang, China; ^2^Mental Health Center, Henan Normal University, Xinxiang, China; ^3^School of Nursing, Southern Medical University, Guangzhou, China; ^4^School of Psychology, Shenzhen University, Shenzhen, China; ^5^Department of Psychology, School of Public Health, Southern Medical University, Guangzhou, China

**Keywords:** psychotherapy style, Psychotherapy Style Scale, therapist style, scale development, psychometric properties

## Abstract

Psychotherapy style is conceptualized as the therapeutic method that a therapist employs while working with clients during treatment. It influences both the therapeutic process and results of therapeutic actions. The present study developed and validated the Psychotherapy Style Scale (PSS). By following a systematic psychometric development process, a three-factor structure of the PSS was identified. Exploratory factor analysis and confirmatory factor analysis provided evidence of multidimensional structure and validity of the PSS. Cronbach’s α suggested that the resulting scale was highly reliable. Criterion validity was also satisfactory, demonstrated by correlations between the scale and criterion validity measures. The PSS has the potential to help better understand therapists’ behavioral characteristics and select the most appropriate therapists for clients who undergo psychotherapy.

## Introduction

Over recent decades, specific characteristics of therapists (i.e., therapist variables) have increasingly been recognized as important factors in psychotherapy (Schiefele et al., 2017). The proportional influence that is attributed to therapist effectiveness was shown to range from 5 to 9% in meta-analyses (Baldwin and Imel, 2013). Therapist variables that are relevant to clinical practice can be divided into demographic and professional variables. Among professional variables, the style of the therapist has received the most attention (Casari et al., 2019). Different therapy styles have been proposed, and some measures to classify them have been suggested.

In an early work in this field, McNair and Lorr (1964) sought to classify psychotherapists according to their characteristic techniques. Based on such research, Analytic–Impersonal–Directive (AID) scales were designed to describe and cluster the conduct and behavior of therapists during interviews with patients. Three dimensions—psychoanalytic, impersonal vs. personal, and directive—were confirmed in these AID scales. The study also suggested that patterns of therapist characteristics were related to their profession, sex, and amount of personal psychotherapy but not to their experience. Although AID scales provided some useful recommendations, most scale items appeared to be more closely related to therapists’ theoretical bias rather than characteristics of therapists’ behavior.

By collecting self-descriptions of general in-therapy behavior from therapists, Rice et al. (1972) identified six different therapeutic styles. The styles included blank screen (e.g., passive and unchanging), paternal (e.g., businesslike and interpretive), transactional (e.g., casual and relationship-oriented), authoritarian (e.g., theory-oriented and persistent), maternal (e.g., explanatory and supportive), and idiosyncratic (e.g., critical and unspontaneous). These six factors accounted for 57.4% of the total variance. The study indicated that experienced and inexperienced therapists as a group had different personal therapeutic styles. Although the classification of therapist styles was a unique perspective, there was a lack of an initial hypothesis, and the generation of items was not entirely clear. Furthermore, the idiosyncratic factor reflected some paradoxical features, such as talkative yet non-provocative. Finally, the sample was composed primarily of therapists who were still in training and lacked statistical power given its small size.

Howard et al. (1986) proposed that application of the social leadership model to the psychotherapy domain was natural. They conceptualized therapist style along the dimensions of direction and support and developed the Therapist Style Inventory Questionnaire. This measure requires therapists to indicate their actual behavior in 12 scenarios. According to varying scores on the two dimensions, therapists were classified into four styles: telling (high direction/low support), teaching (high direction/high support), supporting (low direction/high support), and delegating (low direction/low support). By answering these 12 questions, therapists could derive a sense of their own basic therapeutic style and their style flexibility and range. However, the instrument was more of a tool that allowed the detection of actions that therapists would choose in some situations rather than a scale with distinguishable psychometric properties.

Beutler et al. (1994) published a definitive review on therapist effects. They categorized therapeutic styles as observable states that can be potentially identified by procedures that are independent of the therapist. They found that when therapists and clients have compatible styles of interacting, therapy will likely move in a positive direction. Therapist styles that were postulated to be relevant to client-treatment matching included the therapist’s attempts to increase or decrease the patient’s level of emotional arousal by focusing on affect, with a within-session focus on insight, behavioral change, and directiveness of the therapist’s interventions (Beutler et al., 2012, 2013). Although these styles help us understand the impact of the therapist’s behavior on the client, there is still a lack of a clear distinction between therapy style and therapy orientation.

Further efforts to explore the effects of therapist style were made by Fernández-Álvarez et al. (2003). They described the personal style of the therapist as “the set of characteristics that each therapist applies in every therapeutic situation.” The Personal Style of the Therapist Questionnaire (PST-Q) was constructed according to these authors’ hypotheses. The PST-Q assesses five dimensions of bipolar functions: instructional (flexibility/rigidity), expressive (distance/closeness), engagement (lesser degree/greater degree), attentional (broad focused/narrow focused), and operative (spontaneous/planned). Some empirical studies established a relationship between PST-Q scores and personality factors, the type of clinical client, the duration of treatment, the type of therapy, years of professional experience, and professional training, among others (Castañeiras et al., 2006; Casari et al., 2017, 2019). Although the PST-Q has satisfactory psychometric properties and theoretical validity, its attentional and operative dimensions have a significant factorial weighting on the same factors, and the meaning of the operative dimension is fairly complex.

Overall, most existing measurements focus on therapist characteristics, with only partial consideration of the therapist’s actual behaviors during psychotherapy. As a result, psychotherapy style has not been well distinguished from therapy orientation in these measurements. The PST-Q tried to focus on therapists’ actual behavioral characteristics, but some factors and items reflect behavior of the therapist outside the therapeutic environment. For example, one item in the engagement factor of the PST-Q states, “I think quite a lot about my job even in my spare time,” thus making it difficult to distinguish psychotherapy behavioral style from overall behavioral style.

Therefore, to address some of the shortcomings of existing scales described above, the present study developed a new instrument, the Psychotherapy Style Scale (PSS), to assess therapy style. Our aim was to develop an instrument and determine its validity and reliability. To make the scale better reflect the therapist’s real behavioral characteristics, we first conducted an in-depth interview with experts in psychotherapy. The results of the analysis of this initial interview were then used to form the definition and structure of psychotherapy style. A pool of potential items was then created based on a review of related scales and analysis of the interview. These potential items were evaluated by an expert panel in a Delphi procedure to assess content validity. Item analysis and exploratory factor analysis (EFA) were conducted to explore the latent structure of the items and item reduction. Confirmatory factor analysis (CFA) was conducted to confirm the structure of the scale and evidence of criteria validity and reliability. Our intention was that the instrument should help classify psychotherapists according to their characteristic behaviors, which will help to understand relationships between therapy style and the therapeutic processes, with the overall aim of allowing selection of the most suitable therapist for particular clients.

## Study 1

This study aimed to form the definition and structure of psychotherapy style using in-depth interviews, create a pool of potential items, and evaluate these potential items using Delphi polls.

### Materials and Methods

#### In-Depth Interviews and Qualitative Analysis

##### Participants

Based on a review of the theoretical and empirical literature on therapist styles and related scales, an in-depth interview was conducted. A convenience sample of nine therapists who were leading experts in psychotherapy in Guangdong province, China, were interviewed. The experts were selected based on their extensive experience in psychotherapy in different fields. Of the nine interviewees, four were females and five were male. With regard to practice settings, three were in university psychotherapy centers, three were in hospitals, and three were in private institutions. The interviewees had been in practice for 10–30 years, and their cumulative direct clinical experience ranged from 2000 to more than 10000 h. All of the interviewees indicated that they had been systematically trained in more than two schools of therapeutic approaches, including psychodynamic therapy (9), cognitive behavioral therapy (9), person-centered therapy (7), narrative therapy (4), sandplay therapy (4), family therapy (5), acceptance and commitment therapy (1), and transactional analysis therapy (1).

##### Procedure

The interviewees who had been selected by the study team were sent an email that explained the nature of the study, described the interview protocol, and requested their participation. All of the interviewees agreed to participate by responding an email with their informed consent. The interviews were conducted face-to-face. Each interview lasted approximately 2 h. A semi-structured protocol was used in the interviews. Four questions were asked. (1) “Please describe the general behavioral characteristics that you like to apply in therapy situations.” (2) “Please describe the general behavioral characteristics that other therapists who you known well liked to apply in therapy situation.” (3) “Do you think psychotherapy style is a suitable concept to describe the general behavioral characteristics that therapists like to apply in therapy situations? Please explain why.” (4) “What is psychotherapy style in your opinion? Please give some examples.” Question 4 was not asked if the interviewee did not agree with the existence of a psychotherapy style.

##### Analysis

Data that were collected through the interviews were recorded and analyzed using consensual qualitative research (CQR) (Hill et al., 1997, 2005) by four of the study team members. Five steps were conducted in the CQR process: developing domains, constructing core ideas, conducting cross analyses, examining patterns in the data, and charting the results. The representativeness of the category was determined by how frequently the categories applied to the whole sample. A category that applies to all cases was considered to be general. A category that applied to at least half of the cases was considered typical. A category that applied to either two or three to just less than half of the cases was considered variant. Categories that applied only to one or two cases were dropped because they were not considered to be descriptive of the sample. The results of the analysis were used to form the definition and structure of psychotherapy style. Statements that were rated as general, typical, or variant were selected to be recast as an initial list of possible items, and other statements were dropped.

#### Item Generation

The existing instruments reflecting therapy styles were reviewed to identify potential items for inclusion in the new questionnaire. If an item reflected the definition of therapy style which had been obtained in the in-depth interviews, it was included in the pool of potential items. Some items were also constructed by the study team based on their own clinical experience and understanding of therapy styles.

#### Content Validity Evaluation of the Items

##### Participants

To assess content validity, the potential items were evaluated and modified by an expert panel in a Delphi procedure (McKenna, 1994) over two rounds.

A convenience sample of 30 experts in psychotherapy were invited to participate in the first round. They were sent an email explaining the purpose and main content of the study, the definition and dimensions of psychotherapy style, and what they will do in the Delphi procedure. Twenty-seven experts actually participated by emailing their informed consent, which appeared to be acceptable (Adler and Ziglio, 1996).

The expert panel consisted of 20 women and seven men. Of these, 17 worked in universities, five worked in hospitals, and five worked in private institutions. Regarding their experience, 19 had more than 15 years of experience in psychotherapy. In terms of their theoretical orientation, 16 were integrating therapy, four were psychodynamic therapy, three were cognitive behavioral therapy, three were humanistic therapy, and one was systemic therapy.

Among those experts participating in the first round, 16 took part in the second round. Of these, 10 were women, nine worked in universities, three worked in hospitals, and four worked in private institutions. All the theoretical orientations of the experts were integrating therapy.

##### Procedure and analysis

Experts were asked to evaluate whether the items were relevant and accurate measures of psychotherapy style using a five-point Likert scale. The item-level content-validity index (I-CVI) (Lynn, 1986) was used to evaluate to what extent the experts agreed on the relevance of the items. When there was insufficient agreement on an item’s relevance, the item was removed. Items were also amended according to the experts’ suggestions.

In both rounds, these evaluations and analyses were conducted. The difference between the two rounds is that the first round consisted of a review of the initial version of the questionnaire, but the second consisted of a review of the new version following changes proposed during the previous round. After this stage, items were randomly ordered to create a pilot scale to undergo further psychometric validation.

### Results

#### Item Generation

In the in-depth interviews, all participants agreed that every therapist has a different therapy style. Three themes, namely, the definition, structure, and influential factors relating to therapy style, were identified in the content analysis. All interviewees thought that the psychotherapy style referred to a therapist’s overall manner that the therapist applied in every situation. Seven interviewees thought that psychotherapy style remained stable over time, although it could change slightly. All interviewees considered that the psychotherapy style could lead the therapist to behave in a particular way which made the therapy unique. Referring to the above comments, therapy style was conceptualized as a set of behavioral characteristics that each therapist tends to apply in therapy situation.

In terms of the structure, all interviewees described a kind of therapy style referred to the degree to which the therapists wanted to guide and manage the direction and process of the therapy, which was named directiveness. Seven interviewees described a kind of therapy style referring to the relationship distance between the client and the therapist, which was named closeness. Seven interviewees described another kind of therapy style referred whether the therapist’s behavior was planned or spontaneous, which was named planning. One interviewee described a kind of therapy style referred whether the therapist focused on the past or the future, another interviewee thought flexibility was a kind of therapy style. However, according to the evaluation standards for the representativeness of the category, they were dropped. Finally, three dimensions were constructed: directiveness, closeness, and planning. The demographic characteristics, personality, and professional training background of therapists were proposed to influence therapy style.

On the basis of the content analysis of the descriptions about the interviewees’ own psychotherapy style, the analysis of related instruments, and the items developed by the researchers, an initial pool of 40 items was generated. Of these, Directiveness contained 10 items, Closeness contained 18 items, and Planning contained 12 items.

#### Item Reduction

All the experts who had been invited to participate completed the evaluation in the two Delphi rounds. An I-CVI of 0.78 was the cutoff for an item either to be removed from the instrument or to be preserved (Lynn, 1986). In the first round, eight items were deleted for low representativeness (e.g., “In psychotherapy, I will tell the clients what they need to do”), one item was modified to improve its clarity (“I will avoid emotional involvement” was revised to “I will avoid personal emotion involvement in psychotherapy”), and two items were added according to the experts’ suggestions (“I tend to provide direct advices or guidance to the clients” and “I tend to take the lead when deciding what to do in the psychotherapy”). Then, a new version of the questionnaire containing 36 items has been constructed to be evaluated in the second Delphi round. In the second Delphi round, two items were deleted for low representativeness (“I will express my feelings in psychotherapy” and “I will avoid personal emotion involvement in psychotherapy”). The final version of the PSS comprised 34 items (more information on the Delphi method is available from the authors).

## Study 2

After the two Delphi rounds, a preliminary version of PSS was constructed. This study aimed to further examine what items should be retained through item analysis and explore its validity via EFA.

### Materials and Methods

#### Participants

A total of 235 participants completed the survey (137 females, 98 males). Four different types of workplace were represented in the sample, but the large majority was that of schools (148), followed by social organizations (36), hospitals (33), and other organizations (18). Among the participants, 112 had participated in psychodynamic training, 135 in cognitive behavioral training, 102 in humanism–existentialism training, and 88 in training in other orientations (such as EMDR, family therapy, narrative therapy, and Gestalt therapy). In terms of cumulative direct clinical hours, 136 were between 300 and 500, and 57 were between 501 and 1000. The rest of the therapists’ cumulative direct clinical hours were more than 1000.

#### Procedure

First, questionnaires including the pilot scale and some demographic questions were set up on a professional online survey platform named Wen Juan Xing. Participants could find out the site via a special link. Second, some therapists working at Henan Normal University, Southern Medical University, and Shenzhen University were invited to participate in the survey. Third, some other therapists were invited to take part in by the ones who had completed the survey in order to guarantee the quality of the participants. In this way, more and more therapists completed the survey until the number of the participants met the research requirements.

#### Data Analysis

Correlations were used to conduct item analyses. Items with low item–dimension correlations (*r* < 0.40) were deleted as they may not have a close relevance to the overarching construct of the scale’s dimension. Kaiser–Meyer–Olkin (KMO) and Bartlett’s test of sphericity were used to consider the appropriateness of factor analysis. Then, a series of EFA were conducted to explore the latent structure of the items and item reduction using SPSS 20.0. The promax rotation method was selected due to the nature of the items (Fabrigar et al., 1999). The criteria for determining the number of factors to retain were eigenvalues greater than 1 and factors containing three or more items (Worthington and Whittaker, 2006). The criteria for item reduction (Worthington and Whittaker, 2006) were as follows: (1) items loading strongly (>0.40) onto factors and (2) items that do not cross-load onto two or more factors.

### Results

#### Item Analysis

Based on the item analysis, one item for Directiveness was removed because of its low correlation with the dimension score (*r* = 0.215). Two items for Planning were deleted for the same reason (*r* = 0.334 and *r* = 0.344). The remaining 31 items were significantly and positively correlated with the dimension score and progressed to the next analysis.

#### Factor Analysis

The results of the Bartlett’s test (χ^2^ = 1576.71, *p* < 0.001) and the Kaiser–Meyer–Olkin statistic (KMO = 0.835 > 0.5) both showed that the data were suitable for EFA.

Exploratory factor analysis with the promax rotation method was used. Using an eigenvalue greater than 1, EFA identified six factors with a cumulative extraction sum of squared loadings of 59.166%. However, three factors only contained one item which cross-loaded onto other factors. According to the criteria for factor reduction, those three factors were dropped. Items loading on multiple factors, or with factor load less than 0.4 and improper classification, were also deleted. The same process was repeated to explore the best-fitting latent structure of the scale. After four rounds of exploratory rotation, 17 items were retained, and three factors were obtained. The variance interpretation rate of the three factors was 55.854%. The items of the original Chinese form are in Appendix. Factor loadings are seen in Table 1.

**TABLE 1:** Exploratory factor analysis for factor loadings of the PSS (*n* = 235).

Items	f1	f2	f3
Item7	**0.777**	0.003	0.095
Item4	**0.766**	0.066	0.133
Item17	**0.710**	0.074	0.140
Item13	**0.696**	0.080	0.252
Item1	**0.664**	0.123	0.139
Item10	**0.658**	0.061	0.162
Item8	0.046	**0.802**	0.050
Item5	0.066	**0.788**	0.086
Item11	0.007	**0.753**	0.235
Item17	0.198	**0.694**	0.179
Item2	0.202	**0.651**	0.095
Item14	−0.063	**0.650**	−0.023
Item15	0.040	0.202	**0.771**
Item12	0.217	0.030	**0.730**
Item9	0.292	0.164	**0.722**
Item3	0.289	0.139	**0.687**
Item6	0.048	0.008	**0.684**

*The bold values mean items with a strong loading (0.40 or higher) on one factor.*

The three factors were defined as follows: Directiveness (six items, *M* = 17.82, SD = 0.27, range = 6–30, skewness = −0.05, kurtosis = 0.33), Closeness (six items, *M* = 24.04, SD = 0.22, range = 13–30, skewness = −0.34, kurtosis = 0.29), and Planning (five items, *M* = 15.58, SD = 0.23, range = 8–25, skewness = −0.15, kurtosis = 0.24). These three factors were consistent with the structure of therapy style formed from the in-depth interviews.

## Study 3

The aim of Study 3 was to further investigate the 17-item PSS factor structure and psychometric properties in a new sample. CFA was conducted to test the three-dimension model of the PSS. Evidence for criteria validity and reliability was evaluated. Importantly, psychotherapy reflects a special interaction between therapists and clients. The behavioral characteristics of the therapist are in accordance with how they behave in other forms of interpersonal communication. From this perspective, the International Personality Item Pool-Interpersonal Circumplex (IPIP-IPC) and Revised Neuroticism Extraversion Openness Personality Inventory (NEO-PI-R) were used to evaluate the criterion validity of the PSS.

Previous researchers have proposed that the traits of dominance and warmth are the primary dimensions of interpersonal behavior. The IPIP-IPC is a brief assessment of the two dimensions (Horowitz et al., 1988). In IPIP-IPC, the Assured-Dominant factor reflects if the individual wants to control or dominate the interpersonal relationship in the process of interpersonal communication. A therapist who likes to dominate the interpersonal relationship in daily life will show the similar behavior style during the psychotherapy process. So it was used as the criterion measure of Directiveness. The Warm-Agreeable factor in IPIP-IPC reflects the degree of enthusiasm in the process of personal communication. A therapist who is warm to others will do the same to the clients, so it was used as the criterion measure of Closeness. NEO-PI-R is a measurement of personality domains. In NEO-PI-R, the Order subscale reflects if the person likes to arrange everything carefully (Dai et al., 2004). A therapist who had a high score on the Order subscale will like to arrange the psychotherapy carefully too. So it was used as the criterion measure of Planning.

### Materials and Methods

#### Participants

A total of 468 participants completed the survey (299 females, 169 males). Four different types of practice settings were represented in the sample: hospitals (163), schools (145), social organizations (91), and other organizations (69). In terms of cumulative direct clinical hours, 196 were between 300 and 500, 158 were more than 1000, and 114 were between 501 and 1000.

#### Instruments

Psychotherapy Style Scale (PSS). The PSS contains three dimensions and 17 items as reported in Study 1. Each item was rated on a five-point Likert scale (1 = never like this, 2 = most of the time not like this, 3 = half the time like this, 4 = most of the time like this, and 5 = always like this). The higher the total score for each dimension, the stronger the therapist’s tendency toward this dimension.

The Assured-Dominant factor (PA) and Warm-Agreeable factor (LM) were used as the criterion measures of Directiveness and Closeness. They were selected from a Chinese version of the IPIP-IPC (Horowitz et al., 1988; Hao, 2016). Each contained four items, using a five-point Likert scale (ranging from “very inaccurate” = 1 to “very accurate” = 5). The Cronbach’s alphas for the PA and LM were 0.740 and 0.712, respectively.

The Order subscale from a Chinese version of the NEO-PI-R (Dai et al., 2004) was used as the criterion measure of Planning. The subscale contained eight items, using a five-point Likert scale (ranging from “strongly disagree” = 0 to “strongly agree” = 4). The Cronbach’s alpha for the Order subscale was higher than 0.77.

#### Procedure

Using the same procedure as in Study 2, therapists working at Southern Medical University, Shenzhen University, Henan Normal University, Nanchang University, and Central China Normal University were recruited on a voluntary basis to take part. They also invited peers with whom they were familiar to complete the survey. Participants received instructions and information about how to complete the questionnaires. The answers were recorded anonymously.

#### Data Analysis

First, CFA was conducted using AMOS 7.0 on the 17-item PSS. The indices that were used to evaluate the fit of the model were as follows: the Satorra-Bentler Chi Square divided by degrees of freedom, Comparative Fit Index (CFI), Good Fit Index (GFI), Adjusted Good Fit Index (AGFI), Tucker–Lewis Index (TLI), Normed Fit Index (NFI), Incremental Fit Index (IFI), Root of the Mean Square Residual (RMR), and Root Mean Square Error of Approximation (RMSEA) (Hu and Bentler, 1999). Second, correlational analyses were conducted using SPSS 20.0 to assess criterion validity. Finally, Cronbach’s alpha coefficient was used to analyze the reliability of the scale.

### Results

#### Descriptive Analyses

Descriptive analyses showed that the distributions were reasonably normal (D’Agostino et al., 1990) for all the items, with the skewness values ranging from −0.81 (Item 14) to 0.38 (Item 4), and the kurtosis values ranging from −0.68 (Item 13) to 0.49 (Item 14), respectively. The three dimensions were approximately normally distributed too, that is, Directiveness (*M* = 18.80, SD = 0.21, range = 6–30, skewness = 0.03, kurtosis = 0.17), Closeness (*M* = 23.54, SD = 0.21, range = 6–30, skewness = −0.70, kurtosis = 0.46), and Planning (*M* = 16.44, SD = 0.18, range = 5–25, skewness = −0.15, kurtosis = 0.24).

#### Confirmatory Factor Analysis

**FIGURE 1:**
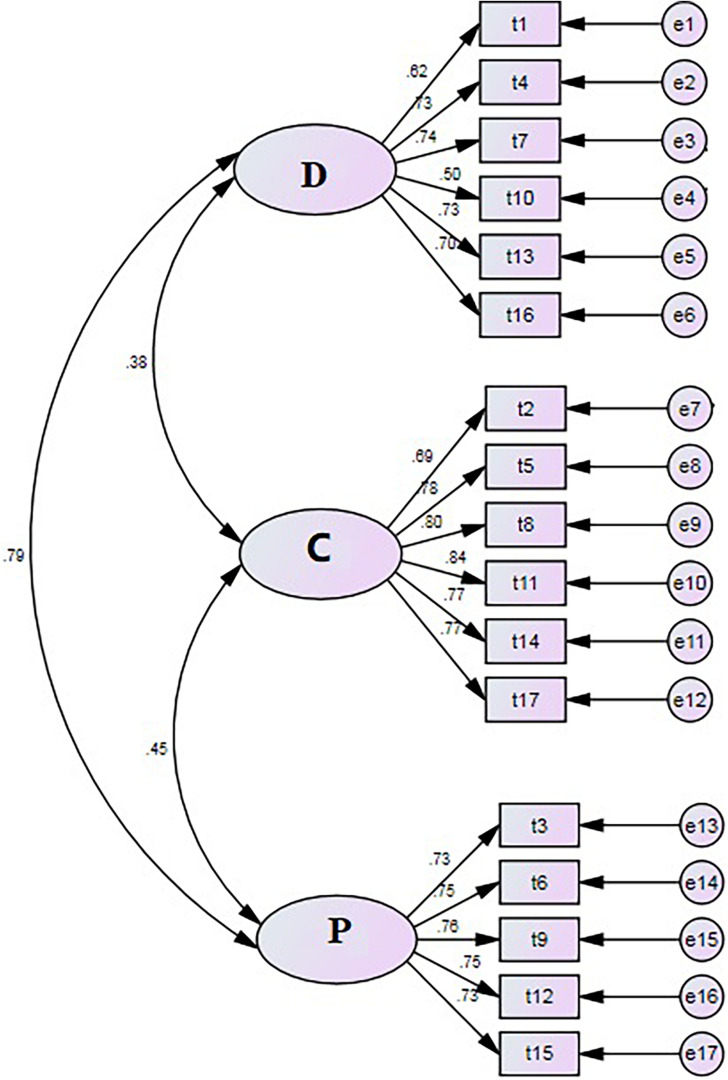
Confirmatory factor analysis model of the PSS (n = 468).

Consistent with the findings of Study 1, the results of the CFA for Sample 2 supported the three-factor model suggested by the results of the EFA with Sample 1 (see Figure 1). The model fit was acceptable, χ^2^ = 309.36, χ^2^/df = 2.762, *p* < 0.001, CFI = 0.951, GFI = 0.928, AGFI = 0.901, TLI = 0.910, NFI = 0.926, IFI = 0.951, RMR = 0.062, RMSEA = 0.062.

#### Criterion Validation

The correlation coefficients between the scores for Directiveness and PA, Closeness and LM, and the Planning and Order subscales were 0.411 (*p* < 0.001), 0.595 (*p* < 0.001), and 0.279 (*p* < 0.001), respectively. These results indicated that the correlation validity of the PSS scores is acceptable.

#### Internal Consistency

The internal consistency coefficients were 0.840, 0.902, and 0.861 for Directiveness scores, Closeness scores, and Planning scores, respectively. This confirmed that the PSS scores have good internal consistency.

## Discussion

The present study developed and validated the PSS in terms of its ability to measure the behavioral characteristics that therapists tend to apply in psychotherapy situations. The final scale contained 17 items and three factors: Directiveness, Closeness, and Planning. The present results indicated that the PSS has a clear factor structure and good psychometric properties. Use of the PSS could help therapists better understand their behavioral characteristics in therapy and help clients select therapists whose behavioral characteristics are consistent with their own preferences and expectations (Zhou and Zhang, 2018).

As described in the Introduction above, therapy style has been the focus of past work. Rice et al. (1972) proposed that although therapists vary their behavior to suit different kinds of clients, they also have a more generally applicable therapy style. In the in-depth interviews, the experts expressed the same view. They agreed that each therapist has a different style and that they could describe their own style and those of other therapists. Therapy style is conceptualized as a habitual way of working that is influenced by personality, training background, and other factors. These conclusions are consistent with the views of Fernandez-Alvarez and colleagues (Casari et al., 2019).

In our work, three dimensions of therapy style (Directiveness, Closeness, and Planning) were identified in the content analysis of the in-depth interviews. Beutler et al. (2011) claimed that some therapies are more directive than others, which can have both positive and negative outcomes. Positive outcomes include clarity, feelings of security, and saving time. Negative outcomes include a decrease in agency, an increase in resistance, and less client self-attribution of improvement (Rautalinko, 2017). Assessing the directiveness of the therapist is very important, especially when considering that some studies suggested that Asian-American clients generally tend to be more willing to remain in treatment when working with directive than with non-directive therapists (Cao, 2009).

Closeness is another important concept that is central to the thinking of many psychotherapists about the processes and goals of their work (Schwartz, 1993). A closer relationship can facilitate talking about difficult issues, evoke target behavior, and augment the effects of various interventions. A genuinely close relationship may exceed the therapy hour, defy control, and evoke unintended responses from the client and clinician (Vandenberghe et al., 2019). Thus, closeness as a working style of the therapist may influence the therapeutic process and evoke different responses from clients.

Fernández-Álvarez et al. (2003) hypothesized that therapeutic style can be studied from two basic perspectives: cognitive and motivational–emotional. Beutler et al. (2013) also proposed that directiveness and closeness are the most strongly representative variables of the therapist. In a study by Cooper and Norcross (2016), therapist directiveness vs. client directiveness and emotional intensity vs. emotional reserve were considered two basic dimensions of the scale to measure clients’ preference for therapist style. Similarly, directiveness and closeness are suggested to be two basic components of the PSS. These two factors appear to map closely onto the dominance and warmth dimensions, respectively, of the interpersonal circumplex (Horowitz et al., 2006). Cooper and Norcross (2016) stated that therapist activities may reflect a broader set of interpersonal relationship approaches. When working with clients, therapists may maintain the style of daily interpersonal communication.

The third dimension of the PSS, Planning, reflects whether the therapist’s choice is spontaneous or planned when working with clients. Baldwin (1974) found that repressors expected more planned activity than spontaneous activity by the therapist. It may be useful to consider some types of therapist profiles based on planning scores to match therapists with clients. Planning had already been considered in the PST-Q. Notably, planning is closely related to directiveness, confirmed not only by Fernández-Álvarez et al. (2003) but also in the EFA in the present study. A difference was found between meaning of the two dimensions in view of practical application, so planning was maintained as the third dimension.

An initial item pool for the PSS was established by revising items that were found in the relevant literature, considering different theoretical perspectives, and analyzing the interviewees’ descriptions of their own style. Two Delphi rounds were used to select and modify the items. EFA and CFA were conducted in two different samples. Overall, the results supported the three-dimensional model of therapy style. Positive correlations were found between PSS dimension scores and criterion validation scale scores. The results indicated that therapy style is closely related to personality of the therapist, and we found that the criterion validity of PSS scores was acceptable. Meanwhile, Cronbach’s α values for the three subscale scores were all well above the criterion (>0.70) for adequate homogeneity, thus demonstrating that the scale scores had good reliability.

Compared with previous measurements of psychotherapy style, the PSS has some advantages. First, the PSS focuses on behavioral characteristics that therapists tend to apply in psychotherapy situations and not therapy orientation. As increasingly more therapists believe their theoretical orientation is integrative, behavioral characteristics rather than therapy orientation can help understand the ways in which a therapist performs therapeutic work. Thus, the PSS will be more effective in such a case. Second, we conducted a review of the theoretical and empirical literature and an in-depth interview with experts in psychotherapy to identify potential items for the PSS. The items were generated from the perspective of clinical practitioners of psychotherapy rather than scientists. Thus, the PSS can better reflect clinical experience. Third, the PSS had more satisfactory psychometric properties than some previous measurements.

The present study also has some important limitations. First, the PSS was developed based on limited samples. The participants were invited by other therapists who also participated in the study. This method of sample acquisition may limit the generalizability of the findings. Further studies should test the three-factor structure of the PSS using larger and multiple samples. Second, the criterion validity of Planning dimension scores was relatively low in the present study. One reason for this may be the lack of proper criterion measures. Third, test–retest reliability was not examined in the present study. Other types of validity, such as convergent and discriminant validity, were not evaluated. Future studies should continue to explore the reliability and validity of PSS scores in larger samples. Fourth, PSS scores may have reflected the preference of therapists for a particular therapy style, but we do not know the extent to which their responses reflect their actual behavior. This will be a central topic for future work.

## Data Availability Statement

The raw data supporting the conclusions of this article will be made available by the authors, without undue reservation.

## Ethics Statement

The studies involving human participants were reviewed and approved by Ethics Committee of Institute of Education, Henan Normal University. Written informed consent for participation was not required for this study in accordance with the national legislation and the institutional requirements.

## Author Contributions

SZ and XZ conceived the research ideas and organized the research. SZ performed the studies, analyzed the data, and drafted and revised the manuscript. YH and DL analyzed the data and interpreted the results. DX drafted and revised the manuscript. All the authors contributed to writing sections of the manuscript and read and approved the submitted version.

## Conflict of Interest

The authors declare that the research was conducted in the absence of any commercial or financial relationships that could be construed as a potential conflict of interest.

## Appendix: Items of the original form of the Psychotherapy Style Scale

指导语：您在心理咨询中是如何工作的？以下是一些描述咨询师工作特点的句子，请根据您在心理咨询中的工作方式，在相应的数字上填，“√”。“1”，代表从不如此，“2”，代表很少如此，“3”，代表有时如此，“4”，代表经常如此，“5”，代表总是如此。

Instruction: What is your actual style with clients in psychotherapy? Here are some sentences describing the behavioral characteristics that the therapists tend to apply in the course of his professional work. Please fill in “√” in the corresponding figures according to your style in the psychotherapy. “1” represents never like this, “2” represents most of the time not like this, “3” represents half the time like this, “4” represents most of the time like this, and “5” represents always like this.

1. 我会引导咨询的方向。(I tend to guide the direction of the psychotherapy.)
2. 我会让来访者感到我的关心。(I tend to make the clients feel my care.)
3. 我的咨询结构性非常强。(My psychotherapy is very structured.)
4. 我会向来访者提出直接的建议或指导。(I tend to provide direct advices or guidance to the clients.)
5. 我会让来访者感到亲近。(I tend to make the clients feel close in psychotherapy.)
6. 在一次会谈中，我不会轻易变更咨询的计划。(I will not easily change the psychotherapy plan in an interview.)
7. 我会教来访者一些应对问题的策略或技巧。(I tend to teach the clients some strategies or skills to cope with problems.)
8. 我会让来访者感到我理解他的感受。(I tend to make the clients feel that I appreciate his/her feelings.)
9. 我倾向于预先明确咨询中的任务。(I tend to clarify the tasks in the psychotherapy in advance.)
10. 我会引导来访者谈论一些关键问题。(I tend to guide the clients to talk about some key issues.)
11. 我会让来访者感受到温暖。(I tend to make the clients feel my warmth.)
12. 一般我的咨询会遵循一个既定程序。(I tend to follow an established procedure in psychotherapy.)
13. 在决定咨询中做些什么时，我起主导作用。(I tend to take the lead when deciding what to do in the psychotherapy.)
14. 我会设身处地理解来访者的感觉。(I tend to put myself in the clients’ shoes and consider for their feeling.)
15. 我会尽早制定整体工作方案。(I tend to create a overall work program as soon as possible.)
16. 我会告诉来访者需要怎么做才能解决他的问题。(I tend to tell the clients what needs to be done to solve his/her problem.)
17. 我会让来访者感受到我的友好和热情。(I tend to make the clients feel my friendliness.)
